# Supplementary material for: DMRforPairs: identifying Differentially Methylated Regions between unique samples using array based methylation profiles
Source: BMC Bioinformatics. 2014 May 15;15:141. doi: 10.1186/1471-2105-15-141 (PMC4046028; doi:10.1186/1471-2105-15-141)
Supplement: Additional file 2 — DMRforPairs output for the comparison of A431-MCF7 and NA17018-NA17105. Please start from the HTML files in each folder. Available via the BMC Bioinformatics website. [file 1471-2105-15-141-S2.zip › 1394847754114233_MOESM2_ESM/A431_MCF7/figures/22360.pdf]

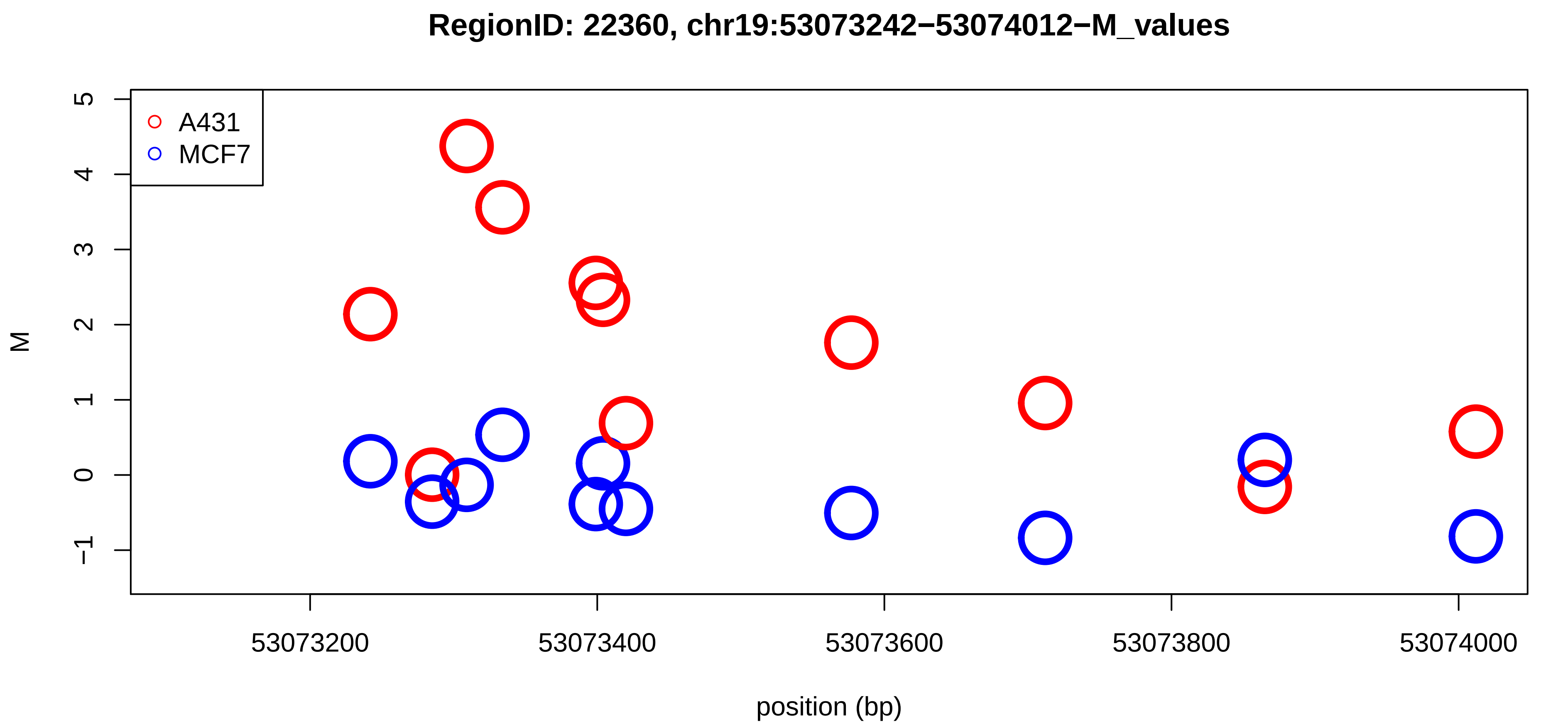
RegionID: 22360, chr19:53073242−53074012−M_values
A431
MCF7
M
5
4
3
2
1
0
−1
53073200
53073400
53073600
53073800
53074000
position (bp)

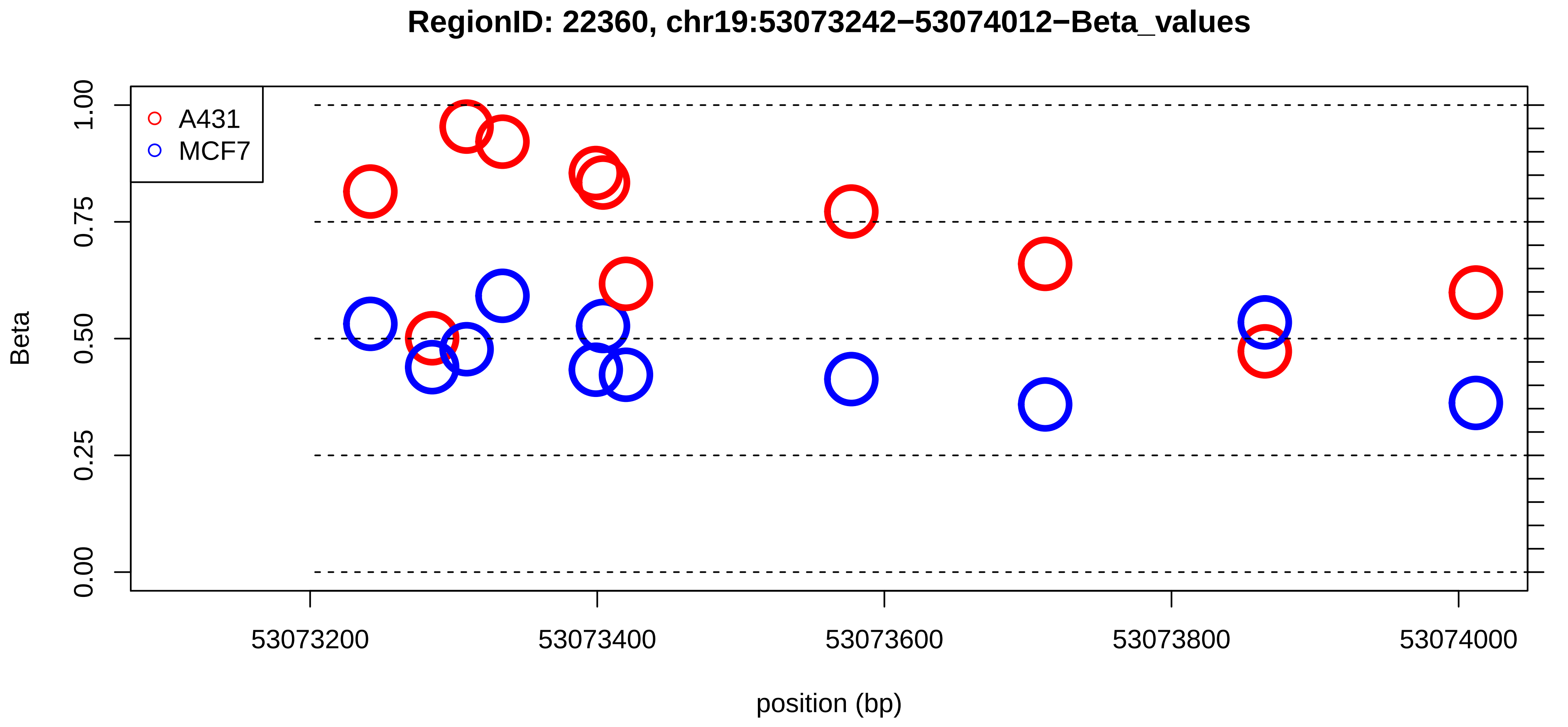
RegionID: 22360, chr19:53073242−53074012−Beta_values
A431
MCF7
Beta
1.00
0.75
0.50
0.25
0.00
53073200
53073400
53073600
53073800
53074000
position (bp)

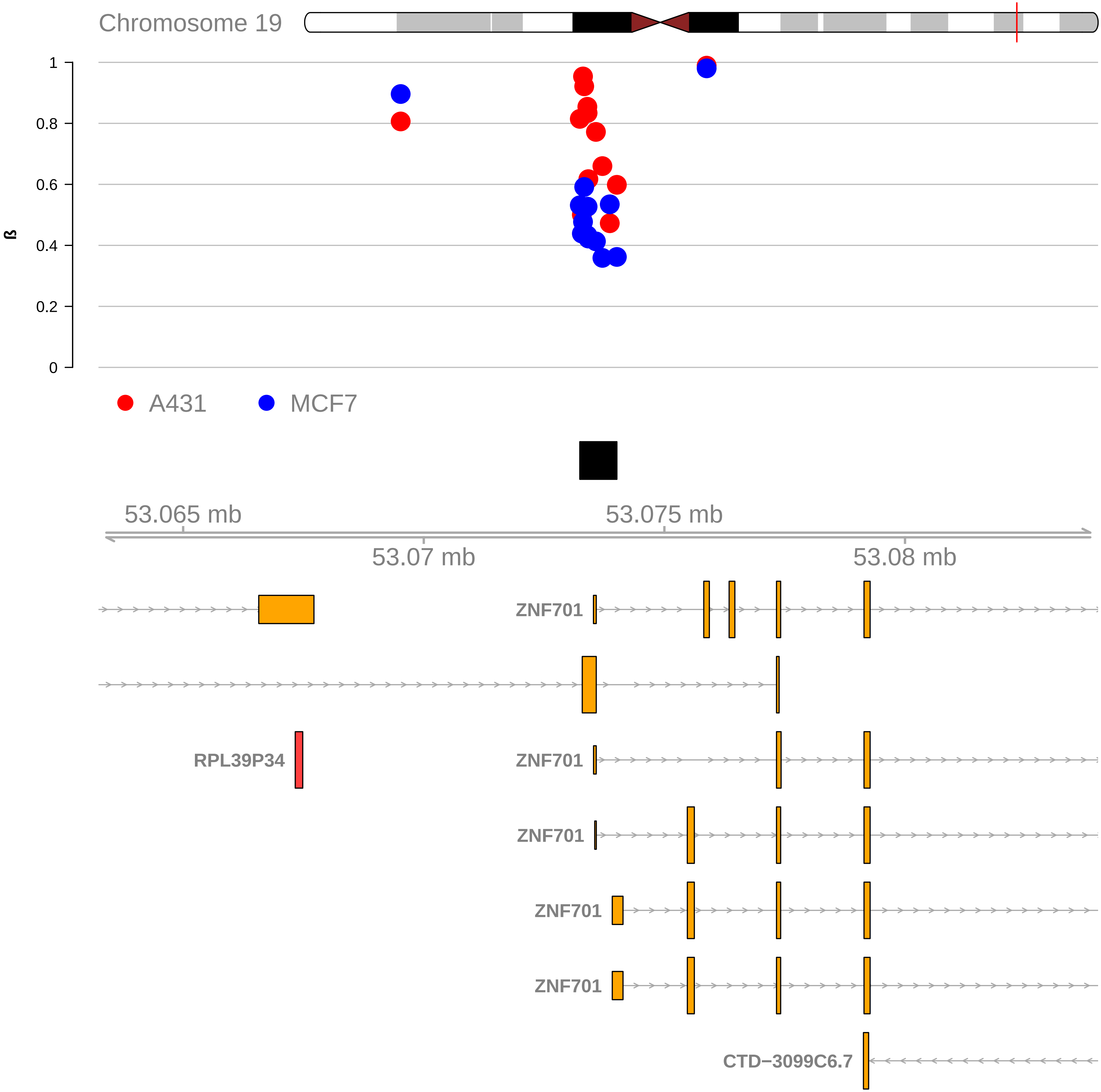

Chromosome 19
1
0.8
0.6
0.4
0.2
0
ß
A431
MCF7
53.065 mb
53.075 mb
53.07 mb
53.08 mb
ZNF701
RPL39P34
ZNF701
ZNF701
ZNF701
ZNF701
CTD−3099C6.7
